# Implementation of the who multimodal hand hygiene improvement strategy in selected wards of Asella Teaching Hospital, Ethiopia

**DOI:** 10.1186/2047-2994-4-S1-P153

**Published:** 2015-06-16

**Authors:** F Pfäfflin, N Schmidt, TB Tufa, T Feldt

**Affiliations:** 1 Klinik für Gastroenterologie, Hepatologie und Infektiologie, Universitätsklinikum Düsseldorf, Düsseldorf, Germany; 2 Hirsch-Institute of Tropical Medicine, Asella, Ethiopia; 3 Institute of Tropical Medicine and International Health, Charité - Universitätsmedizin Berlin, Berlin, Germany

## Introduction

The burden of health-care associated infections (HAI) in low-income countries is high. Adequate hand hygiene is considered the most effective measure to reduce the transmission of nosocomial pathogens.

## Objectives

To assess compliance with hand hygiene and perception and knowledge about hand hygiene before and after the implementation of the hand hygiene campaign

## Methods

The study is carried out in selected wards of ATH (gynaecology, obstetrics, paediatrics, and neonatology).

Compliance with hand hygiene during routine patient care is measured before and after the intervention, which is a four-day workshop accompanied by provision of hand hygiene products and posters emphasizing the importance of hand hygiene. Health-care workers’ (HCW) perception and knowledge about hand hygiene is assessed before and after the intervention. HCWs are divided into two broad professional categories: (I) nurse / midwife / health officer / nurse, midwife, health officer student, (II) medical doctor / intern.

Compliance at baseline and follow-up overall and for the different professional categories and wards will be compared with ξ^2^ tests. Hand hygiene knowledge questionnaire scores will be calculated as the sum of correct answers. Results will be indicated as medians and will be assessed by Wilcoxon rank-sum test.

## Results

We observed a total of 2464 hand hygiene opportunities during 2923 minutes of observation at baseline. Compliance overall was 1.5%. Compliance for professional category I and II was 1.7 and 1.4%, respectively. Compliance on the neonatology ward was 4.1% whereas compliance was less than 1% on the other three wards. The median score for hand hygiene knowledge overall was 13 (interquartile range (IQR) 10-15) at baseline. For professional category I and II, median scores were 12 (IQR 9.3-14) and 14 (IQR 11-16), respectively. The impact of HAI on a patient’s clinical outcome and the effectiveness of hand hygiene in preventing HAI were judged to be high or very high by 73.8% and 96.7% of the HCW, respectively.

## Conclusion

Compliance with hand hygiene at baseline was low in selected wards of ATH and was lower than baseline values in similar settings.

## Disclosure of interest

None declared.

